# Acetylbinankadsurin A Decreases Macrophage Glycolysis and Pro-Inflammatory Phenotype Polarization via Inhibiting HIF-1α to Alleviate Hepatic Fibrosis in Mice

**DOI:** 10.3390/molecules30234571

**Published:** 2025-11-27

**Authors:** Qiang Yao, Wangxia Peng, Huaguan Lu, Yupei Yang, Ziti Rao, Xin Xie, Dan Huang, Wei Wang, Jianye Yan, Jianjun Liu

**Affiliations:** 1 Academy of Chinese Medical Sciences, Hunan University of Chinese Medicine, Changsha 410208, China; 2 School of Pharmacy, Hunan University of Chinese Medicine, Changsha 410208, Chinawangwei402@hotmail.com (W.W.)

**Keywords:** hepatic fibrosis, Acetylbinankadsurin A, macrophage, HIF-1α, glycolysis

## Abstract

Hepatic fibrosis is a prevalent pathological process of chronic liver injury, which can progress to cirrhosis and hepatocellular carcinoma, representing a major cause of mortality in patients with chronic liver disease. *Kadsura coccinea* (Lem.) A. C. Smith possesses pharmacological properties, including antitumor and anti-inflammatory effects, and is primarily used to treat rheumatism, hepatotoxicity injury, and chronic hepatitis. Acetylbinankadsurin A (ACBA) is a natural compound extracted from the roots of *Kadsura coccinea*. However, there have been few studies on the pharmacological activity of ACBA. This study aimed to investigate whether ACBA decreases macrophage glycolysis and pro-inflammatory phenotype polarization by inhibiting HIF-1α to alleviate hepatic fibrosis in mice. In this study, CCl_4_-induced mouse liver fibrosis models and lipopolysaccharide (LPS)-induced THP-1 monocytic cell lines were utilized to simulate macrophage polarization. Techniques such as Western blotting and immunofluorescence were applied to analyze macrophage glycolysis and phenotypes. Our findings revealed that ACBA alleviated CCl_4_-induced hepatic fibrosis in mice and suppressed LPS-induced M1 macrophage polarization. We observed that ACBA significantly reduced the expression of HIF-1α and macrophage glycolysis in liver fibrosis tissue and LPS-induced M1 macrophages. Furthermore, molecular docking, molecular dynamics simulations, and SPR assays demonstrated that there are three sites on the HIF-1α amino acid residues that can stably bind with ACBA in vitro. In conclusion, these results suggest that ACBA inhibits the activity of HIF-1α, thereby decreasing macrophage glycolysis and the pro-inflammatory phenotype, which alleviates hepatic fibrosis in mice.

## 1. Introduction

Hepatic fibrosis (HF) is a pathological process of chronic liver disease caused by various etiologies, such as viral infections, alcoholic or non-alcoholic steatohepatitis, autoimmune disorders, and genetic predispositions, which is characterized by the persistent activation and differentiation of hepatic stellate cells (HSCs) into myofibroblasts, leading to excessive synthesis of extracellular matrix (ECM) and the fibrotic scar formation to displace the normal hepatic structures [1,2]. If not intervened in time, the HF can eventually develop into cirrhosis or hepatocellular carcinoma, which is considered to be an important cause of mortality in chronic liver disease [3]. Consequently, it is very necessary to develop effective anti-fibrotic drugs and therapeutic strategies.

Numerous studies have demonstrated that hepatic macrophages play a pivotal role in regulating the activation of HSCs and determining the initiation, progression, and resolution of HF [4,5]. The macrophages are highly heterogeneous and plastic, displaying distinct phenotypes and functions in response to the various stimulating conditions. Based on their functional characteristics, the macrophages are classified into two basic phenotypes: M1 and M2. M1 macrophages are activated by LPS or INF-γ, can secrete the pro-inflammatory cytokines such as TNF-α and IL-6, and promote the liver inflammation and fibrosis progression. In contrast, the M2 macrophages are stimulated by IL-4 and IL-13, and release anti-inflammatory cytokines, including TGF-β and IL-10, to reduce liver inflammation and promote tissue repair [6]. Therefore, hepatic macrophages are of significant therapeutic interest due to their central role in normal tissue homeostasis and their dual functions in promoting and inhibiting fibrosis, making them attractive targets for therapeutic intervention in experimental animal models and clinical trials [7,8].

Increasing evidence has emphasized that metabolic reprogramming plays an important role in macrophage polarization, and enhanced glycolysis is considered a key feature of inflammatory macrophages. HIF-1α, a mediator of cellular adaptive responses to hypoxia, facilitates metabolic reprogramming from oxidative phosphorylation to glycolysis by upregulating key glycolytic enzymes, such as PFKFB3, and plays a critical role in determining cellular function and cell fate determination [9,10,11,12]. As is known, the stability of the HIF-1α protein depends on the PAS domain within its N-terminal region [13]. The PAS domain is subdivided into PAS-A and PAS-B subdomains, and is necessary for the formation of heterodimerization between HIF-1α and HIF-1β, which ensures their effective binding and regulates the transcription of hypoxia-responsive genes [11,14]. Thus, mutations or impairment of the PAS-B domain can decrease the stability of HIF-1α and various functional responses to hypoxia.

Currently, botanical drugs and phytochemical compounds are receiving increasing attention as adjuvant therapies for liver fibrosis [15,16]. *Kadsura coccinea* (Lem.) A. C. Smith, named as “Heilaohu” in the Tujia ethnomedicine in China, is a medicinal plant of ethnomedicine belonging to the Schisandra genus in the Schisandra family [17]. *Kadsura coccinea* possesses pharmacological properties, including antitumor and anti-inflammatory effects, and is primarily used to treat rheumatism, chronic gastritis, hepatotoxicity injury, and chronic hepatitis [18,19,20]. Acetylbinankadsurin A (ACBA), a natural biphenylcyclooctadiene lignan compound, was extracted from the roots of *Kadsura coccinea* [21]. Currently, there have been few studies on the pharmacological activity of ACBA. However, our previous study results suggested that ACBA significantly ameliorates CCl_4_-induced hepatic fibrosis in mice [22], but its mechanism remains unclear.

The purpose of this study was to investigate whether ACBA could target HIF-1α to inhibit hepatic M1 macrophages, thereby alleviating LPS-induced liver fibrosis in mice. Both in vivo and in vitro experiments demonstrated that ACBA inhibits HIF-1α-mediated glycolysis and M1 macrophage polarization. Additionally, molecular docking, molecular dynamics simulations and SPR assays have shown stable binding between ACBA and HIF-1α. Consequently, ACBA may be a potential therapeutic agent for liver fibrosis by suppressing pro-inflammatory macrophages.

## 2. Results

### 2.1. ACBA Effectively Alleviates CCl_4_-Induced Inflammation and Hepatic Fibrosis in Mice

**Figure 1:**
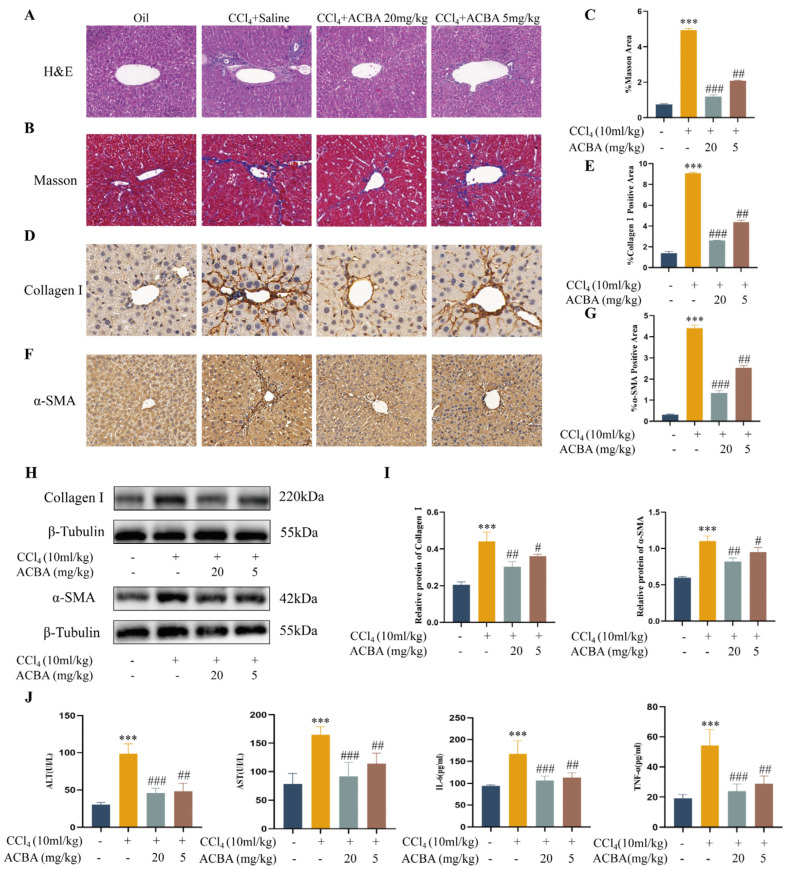
ACBA alleviates the CCl_4_-induced hepatic fibrosis in mice. (**A**) H&E staining of liver tissue, 50×. (**B**) Masson’s trichrome staining of liver tissue, 50×. (**C**) Statistical analysis of collagen fiber deposition areas (*n* = 3). (**D**) Immunohistochemistry staining of Collagen I in liver tissue, 50×. (**E**) Statistical analysis of Collagen I-positive areas (*n* = 3). (**F**) Immunohistochemistry staining of α-SMA in liver tissue, 50×. (**G**) Statistical analysis of α-SMA-positive areas (*n* = 3). (**H**) Western blot analysis of Collagen I and α-SMA. (**I**) Statistical analysis of Collagen I and α-SMA protein expression levels (*n* = 3). (**J**) Levels of ALT, AST, IL-6, and TNF-α (*n* = 5). Data are expressed as mean ± standard deviation. Compared to the control group, *** *p* < 0.001. Compared to the CCl_4_ group, ### *p* < 0.001, ## *p* < 0.01, and # *p* < 0.05.

In this study, two doses of ACBA (20 mg/kg and 5 mg/kg) were used to treat the CCl_4_-induced mouse liver fibrosis models. H&E staining showed that compared to the control group, liver tissue exhibited more necrotic areas, significantly increased local inflammatory cell infiltration, and fibrous septa in the model group. In contrast, ACBA treatment diminished the pathological damage and inflammatory cell infiltration in hepatic tissues (Figure 1A). Masson staining demonstrated that normal liver tissue had no collagen deposition, while extensive collagen accumulation was observed in the central veins and portal tracts in the model group’s liver. Compared with the model group, ACBA treatment notably reduced collagen deposition in liver tissue (Figure 1B,C). Immunohistochemistry staining revealed that normal liver tissue had minimal expression of Collagen I and α-SMA, whereas the model group exhibited high levels. Notably, ACBA treatment decreased the expression of Collagen I and α-SMA in the liver tissues (Figure 1D–G). Western blot analysis confirmed the expression patterns of Collagen I and α-SMA in liver tissue (Figure 1H,I). Additionally, ELISA assays were performed to measure the levels of ALT, AST, IL-6, and TNF-α in the serum of mice. The results demonstrated that ACBA treatment significantly reduced the ALT, AST, and inflammatory factors IL-6 and TNF-α (Figure 1J). These findings indicated that ACBA treatment effectively alleviates hepatic inflammation and the progression of hepatic fibrosis in CCl_4_-induced mouse liver fibrosis models.

### 2.2. ACBA Inhibits M1 Macrophage Polarization in CCl_4_-Induced Mice Hepatic Fibrosis Tissue

**Figure 2:**
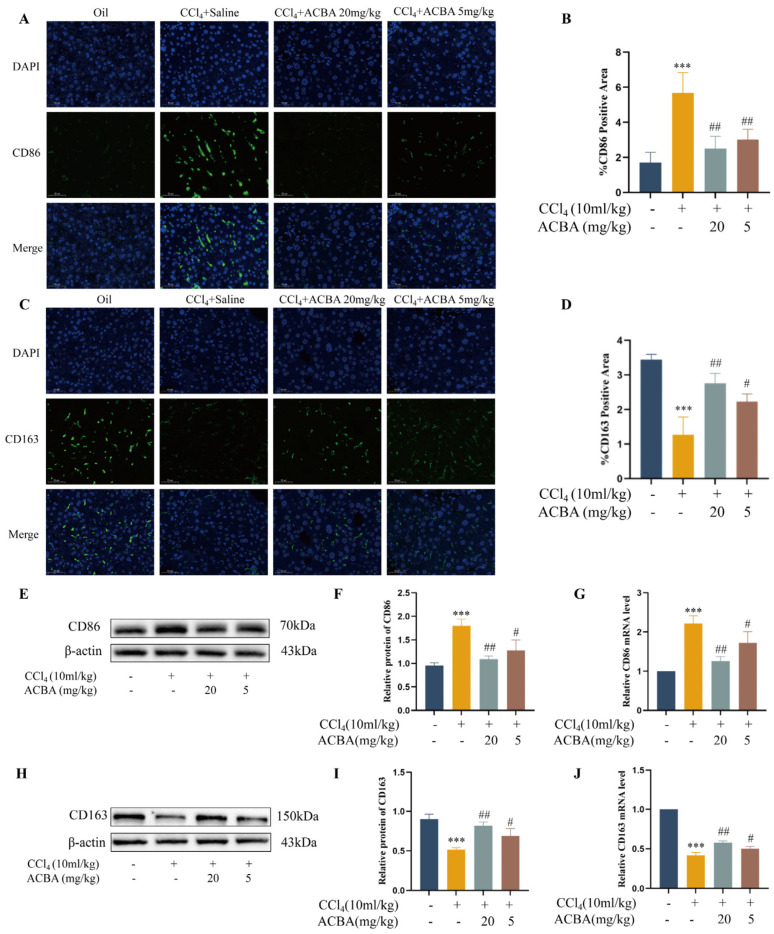
ACBA inhibits M1 macrophage polarization in CCl_4_-induced mouse liver tissue. (**A**) Immunofluorescence staining of CD86 in liver tissue. (**B**) Statistical analysis of CD86-positive areas (*n* = 3). (**C**) Immunofluorescence staining of CD163 in liver tissue. (**D**) Statistical analysis of CD163-positive areas (*n* = 3). (**E**) Western blot analysis of CD86. (**F**) Statistical analysis of CD86 protein expression levels (*n* = 3). (**G**) RT-qPCR analysis of CD86 mRNA levels (*n* = 3). (**H**) Western blot analysis of CD163. (**I**) Statistical analysis of CD163 protein expression levels (*n* = 3). (**J**) RT-qPCR analysis of CD163 mRNA levels (*n* = 3). Data are expressed as mean ± standard deviation. Compared to the control group, *** *p* < 0.001. Compared to the CCl_4_ group, ## *p* < 0.01, and # *p* < 0.05.

During the process of HF, excessive pro-inflammatory M1 macrophages exacerbate hepatic inflammation and activate HSCs to promote collagen production through the TGF-β signaling pathway [23]. In contrast, anti-inflammatory M2 macrophages reduce hepatic inflammatory responses and facilitate tissue repair [24]. Recent studies have identified hepatic macrophages as a crucial target for anti-fibrotic therapy [25]. Consequently, we investigated the effects of ACBA treatment on hepatic macrophage phenotypes. Immunofluorescence staining results showed that CD86, a marker of M1 macrophages, was rarely expressed in the liver tissue of the control group, while CD86 expression increased significantly in the liver tissue of the model group. Compared with the model group, the ACBA treatment group showed significantly reduced CD86 expression in the liver tissue (Figure 2A,B). Conversely, the expression of CD163, a marker of M2 macrophages, was higher in normal liver tissue, and significantly decreased in model liver tissue, while ACBA treatment increased the expression of CD163 in liver tissue (Figure 2C,D). Western blot protein detection (Figure 2E,F,H,I) and RT-qPCR mRNA analysis (Figure 2G,J) also produced similar results. These results demonstrate that ACBA can inhibit pro-inflammatory macrophage polarization in CCl_4_-induced mouse liver fibrosis.

### 2.3. ACBA Inhibits Pro-Inflammatory Phenotype and Function in LPS-Induced THP-1 Cells

**Figure 3:**
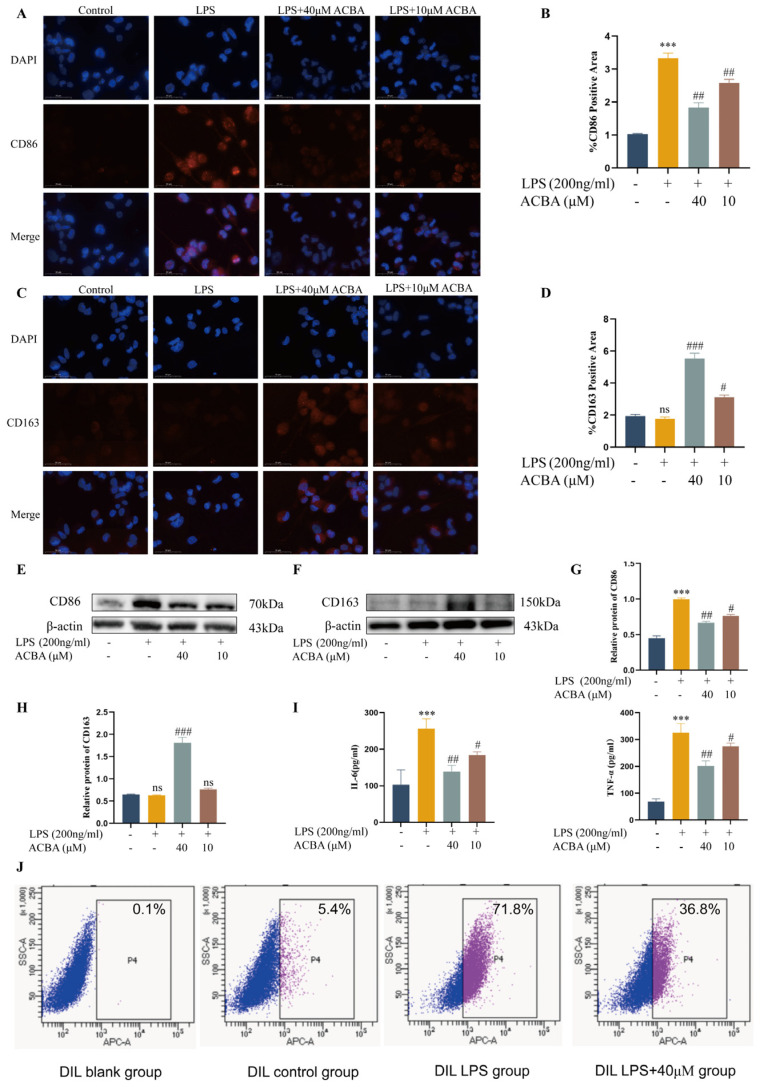
ACBA inhibits the M1 macrophage polarization in LPS-induced THP-1 cells. (**A**) Immunofluorescence staining of CD86 in THP-1 cells. (**B**) Statistical analysis of CD86-positive cells (*n* = 3). (**C**) Immunofluorescence staining of CD163 in THP-1 cells. (**D**) Statistical analysis of CD163-positive cells (*n* = 3). (**E**) Western blot analysis of CD86. (**F**) Western blot analysis of CD163. (**G**) Statistical analysis of CD86 protein expression levels (*n* = 3). (**H**) Statistical analysis of CD163 protein expression levels (*n* = 3). (**I**) Level of IL-6 and TNF-α (*n* = 5). (**J**) Flow cytometry analysis of CD86+-positive cells. Data are expressed as mean ± standard deviation. Compared to the control group, *** *p* < 0.001; ns, not significant. Compared to the LPS group, ### *p* < 0.001, ## *p* < 0.01, and # *p* < 0.05.

To verify that ACBA inhibits M1 macrophage polarization, we treated LPS-induced THP-1 cells with ACBA at two concentrations of 40 μmol/L and 10 μmol/L. Immunofluorescence staining results indicated that LPS significantly increased CD86 expression in THP-1 cells, whereas ACBA treatment significantly inhibited this increase (Figure 3A,B). Although LPS did not significantly alter CD163 expression in THP-1 cells, ACBA treatment notably enhanced the expression of CD163 in THP-1 cells, particularly in the group treated with 40 μmol/L ACBA (Figure 3C,D). Similar results were obtained by Western blot analysis (Figure 3E–H). Furthermore, we assessed the levels of inflammatory factors in the cell culture supernatant of each group. ELISA results demonstrated that LPS markedly elevated the levels of IL-6 and TNF-α in the culture supernatant of THP-1 cells, whereas ACBA treatment reduced the secretion of IL-6 and TNF-α (Figure 3I). Additionally, we employed flow cytometry to analyze the proportion of M1 macrophages in LPS-induced THP-1 cells with 40 μmol/L ACBA. The results showed that 71.8% of LPS-induced THP-1 cells were CD86-positive cells, compared to 36.8% in the ACBA-treated group (Figure 3J). These results suggest that ACBA inhibits the M1 macrophage polarization in LPS-induced THP-1 cells.

### 2.4. ACBA Decreases the Expression of PFKFB3 in CCl_4_-Induced Mouse Hepatic Fibrosis Tissue

**Figure 4:**
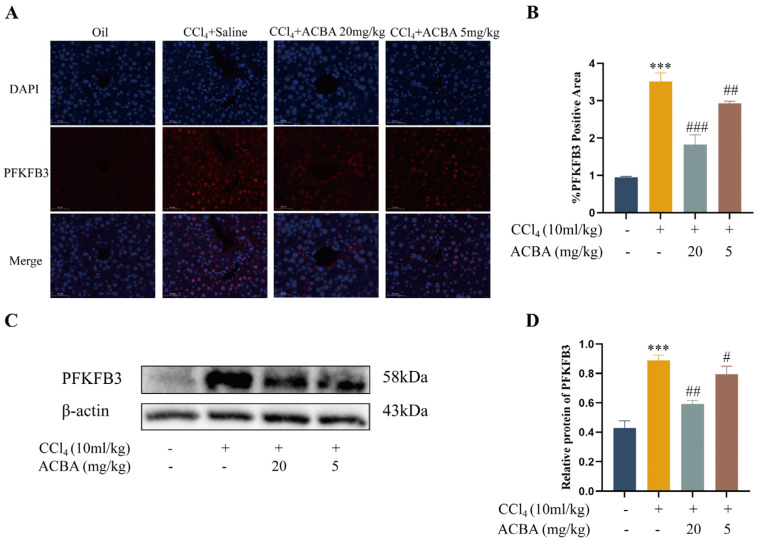
ACBA reduces PFKFB3 expression in CCl_4_-induced mouse liver tissue. (**A**) Immunofluorescence staining of PFKFB3 in liver tissue. (**B**) Statistical analysis of PFKFB3-positive areas (*n* = 3). (**C**) Western blot analysis of PFKFB3. (**D**) Statistical analysis of PFKFB3 protein expression levels (*n* = 3). Data are expressed as mean ± standard deviation. Compared to the control group, *** *p* < 0.001. Compared to the CCl_4_ group, ### *p* < 0.001, ## *p* < 0.01, and # *p* < 0.05.

PFKFB3 plays a crucial role in the glycolytic process and serves as a critical regulator of macrophage glycolysis, contributing to the progression of liver fibrosis [26]. Firstly, we analyzed the expression patterns of PFKFB3 in mouse liver tissues. Immunofluorescence staining revealed that PFKFB3 expression was low in normal liver tissue but significantly increased in liver fibrosis tissue of the model group. Compared with the model group, PFKFB3 expression was significantly reduced in the liver tissue of the ACBA treatment group (Figure 4A,B). Western blot results also confirm the expression pattern of PFKFB3 (Figure 4C,D). These findings demonstrate that ACBA effectively inhibits PFKFB3 expression in liver tissues of CCl_4_-induced liver fibrosis models.

### 2.5. ACBA Inhibits the Expression of PFKFB3 and Glycolysis Levels in LPS-Induced THP-1 Cells

**Figure 5:**
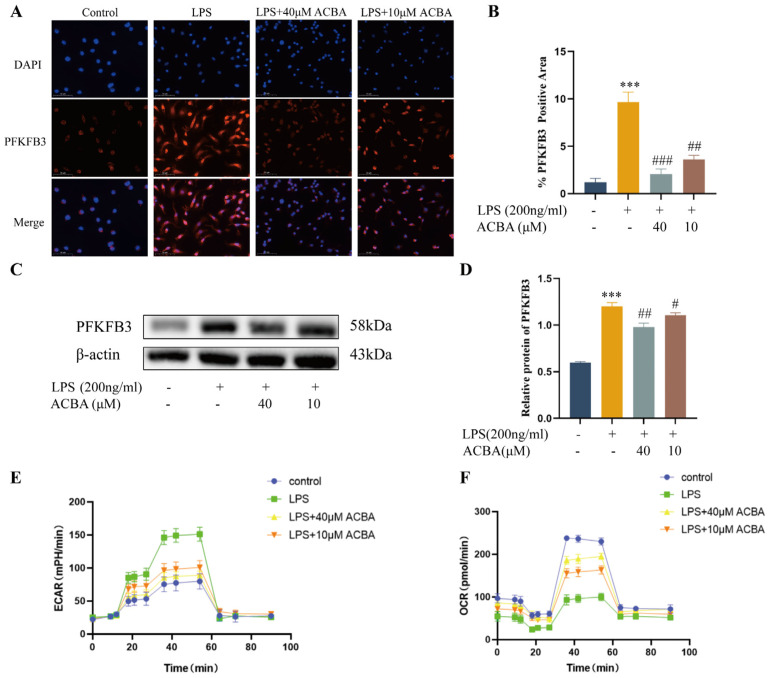
ACBA inhibits the glycolysis in LPS-induced THP-1 cells. (**A**) Immunofluorescence staining of PFKFB3 in THP-1 cells. (**B**) Statistical analysis of PFKFB3-positive cells (*n* = 3). (**C**) Western blot analysis of PFKFB3. (**D**) Statistical analysis of PFKFB3 protein expression (*n* = 3). (**E**) Extracellular acidification rate in THP-1 cells. (**F**) Oxygen consumption rate in THP-1 cells. Data are expressed as mean ± standard deviation. Compared to the control group, *** *p* < 0.001. Compared to the LPS group, ### *p* < 0.001, ## *p* < 0.01, and # *p* < 0.05.

To investigate whether ACBA inhibits PFKFB3 expression and glycolysis in macrophages, we treated LPS-induced THP-1 cells with ACBA at two concentrations of 40 μmol/L and 10 μmol/L. Immunofluorescence staining and Western blot analysis revealed that compared to the control group, LPS significantly increased the expression of PFKFB3 in THP-1 cells. However, treatment with ACBA inhibited the expression of PFKFB3 induced by LPS (Figure 5A–D). Given that ECAR and OCR are critical indicators of glycolytic flux dynamics [27], we further assessed the effects of ACBA on ECAR and OCR in LPS-induced THP-1 cells. The results showed that LPS notably enhanced ECAR in THP-1 cells while diminishing OCR. In contrast, ACBA treatment substantially decreased LPS-induced ECAR but elevated OCR (Figure 5E,F). These results demonstrate that ACBA inhibits the expression of PFKFB3 and the glycolytic level in LPS-induced THP-1 cells.

### 2.6. ACBA Inhibits the Expression of HIF-1α in CCl_4_-Induced Hepatic Fibrosis Tissue in Mice

**Figure 6:**
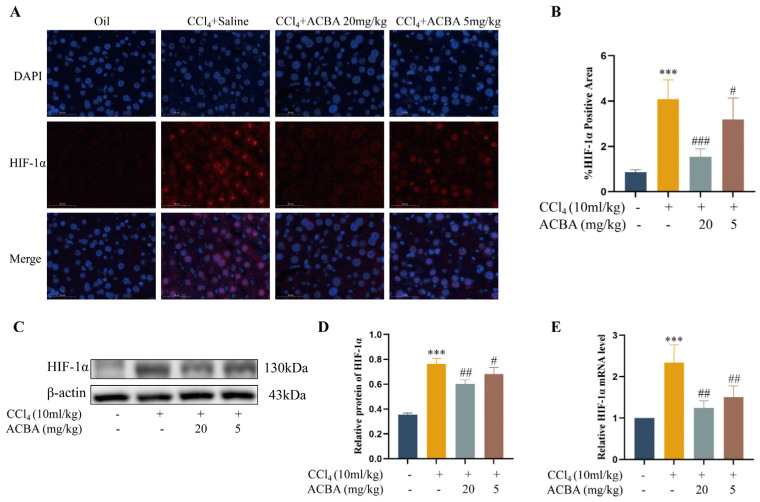
ACBA inhibits the HIF-1α expression in CCl_4_-induced mouse liver tissue. (**A**) Immunofluorescence staining of HIF-1α in liver tissue. (**B**) Statistical analysis of HIF-1α-positive areas (*n* = 3). (**C**) Western blot analysis of HIF-1α. (**D**) Statistical analysis of HIF-1α protein expression levels (*n* = 3). (**E**) RT-qPCR analysis of HIF-1α mRNA levels (*n* = 3). Data are expressed as mean ± standard deviation. Compared to the control group, *** *p* < 0.001. Compared to the CCl_4_ group, ### *p* < 0.001, ## *p* < 0.01, and # *p* < 0.05.

Under hypoxic conditions, macrophage pro-inflammatory activation and glycolysis are significantly enhanced, a process that relies on PFKFB3 and HIF-1α [9,10]. HIF-1α mediates the transcription of PFKFB3, accordingly regulating macrophage glycolysis and pro-inflammatory functions [28,29]. As such, we investigated the effects of ACBA on HIF-1α expression in CCl_4_-induced mouse liver fibrosis tissue. Immunofluorescence staining and Western blot analyses revealed that, in comparison to the control group, HIF-1α expression was significantly elevated in the liver fibrosis tissue of CCl_4_-induced mice. However, ACBA treatment significantly reduced the expression of HIF-1α (Figure 6A–D). In addition, RT-qPCR results also demonstrated that HIF-1α mRNA levels were considerably increased in CCl_4_-induced mouse liver fibrosis tissue, relative to the control group, and ACBA treatment led to a significant decrease in HIF-1α mRNA expression (Figure 6E). These findings indicate that HIF-1α exhibits high expression levels in CCl_4_-induced liver fibrosis tissue, which is consistent with previous reports [30], while ACBA treatment can effectively reduced the HIF-1α expression in hepatic fibrosis tissue.

### 2.7. ACBA Inhibits the Expression of HIF-1α in LPS-Induced THP-1 Cells

**Figure 7:**
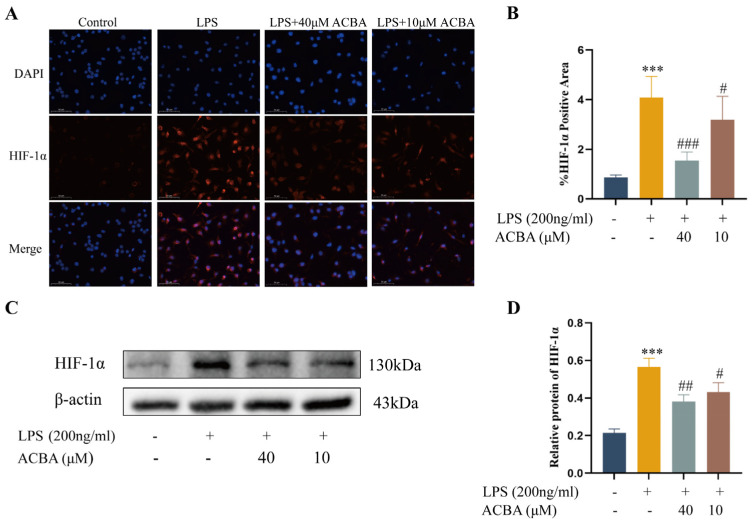
ACBA inhibits the HIF-1α expression in LPS-induced THP-1 cells. (**A**) Immunofluorescence staining of HIF-1α in THP-1 cells. (**B**) Statistical analysis of HIF-1α-positive areas (*n* = 3). (**C**) Western blot analysis of HIF-1α. (**D**) Statistical analysis of HIF-1α protein expression levels (*n* = 3). Data are expressed as mean ± standard deviation. Compared to the control group, *** *p* < 0.001. Compared to the LPS group, ### *p* < 0.001, ## *p* < 0.01, and # *p* < 0.05.

Furthermore, we treated LPS-induced THP-1 cells with two concentrations of ACBA (40 μmol/L and 10 μmol/L) to investigate whether ACBA could regulate HIF-1α expression in macrophages. Immunofluorescence staining showed that, compared with the control group, HIF-1α expression in LPS-induced THP-1 cells was significantly enhanced, while ACBA treatment significantly inhibited HIF-1α expression (Figure 7A,B). Western blot analysis produced similar results (Figure 7C,D). These results suggested that ACBA inhibited the expression of HIF-1α in LPS-induced THP-1 cells.

### 2.8. Analysis of ACBA and HIF-1α Interaction

**Figure 8:**
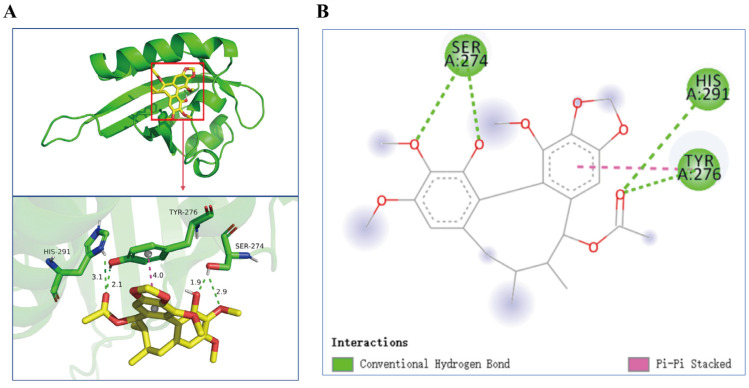
Docking modes of ACBA and HIF-1α. (**A**,**B**) Visualization of the interaction between ACBA and HIF-1α. Green lines indicates hydrogen bonding interactions, and pink lines indicates π-π stacked.

Molecular docking was performed to investigate potential binding modes and interactions between ACBA and HIF-1α. The results from the Autodock Vina 1.5.6 software for molecular docking simulations indicated that the lowest affinity score between ACBA and HIF-1α was −6.0 kcal/mol (Table 1). Upon analyzing the binding modes with the Pymol 3.1 and Discovery Studio Visualizer 2019 software, we observed that ACBA forms four hydrogen bonds with the amino acid residues SER-274, TYR-276, and HIS-291 of HIF-1α, and reveals π-π stacking with TYR-276 (Figure 8A,B).

**Figure 9:**
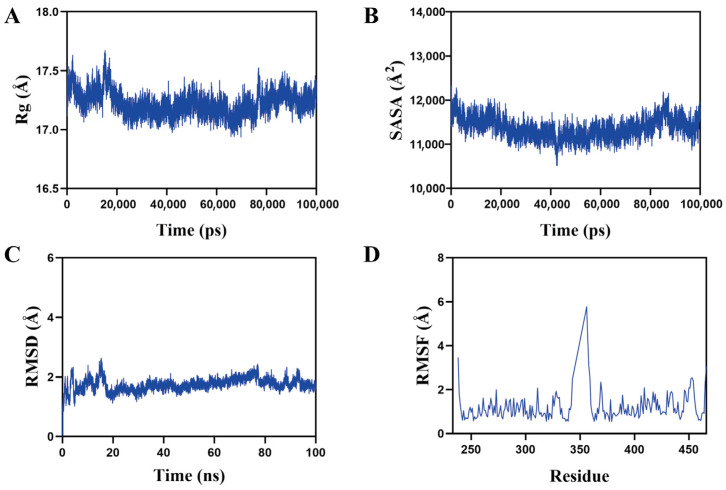
Results of molecular dynamics simulations of ACBA and HIF-1α. (**A**) Results of Rg between ACBA and HIF-1α. (**B**) Results of SASA between ACBA and HIF-1α. (**C**) Results of RMSD between ACBA and HIF-1α. (**D**) Results of RMSF between ACBA and HIF-1α.

Then, molecular dynamics simulations were utilized to evaluate the stability of the ACBA-HIF-1α complex. The radius of gyration (Rg) results showed that the radius generally has a downward trend between 17.0 Å and 17.5 Å (Figure 9A). The solvent-accessible surface area (SASA) of the complex is mostly between 11,000 Å^2^ and 12,000 Å^2^ (Figure 9B). These findings revealed that both the Rg and SASA of the complex system displayed minor variations during the simulation. The root mean square deviation (RMSD) values for the system predominantly were clustered near 2.1 Å. (Figure 9C). The root mean square fluctuation (RMSF) variations during the entire simulation were primarily under 2 Å (Figure 9D).

**Figure 10:**
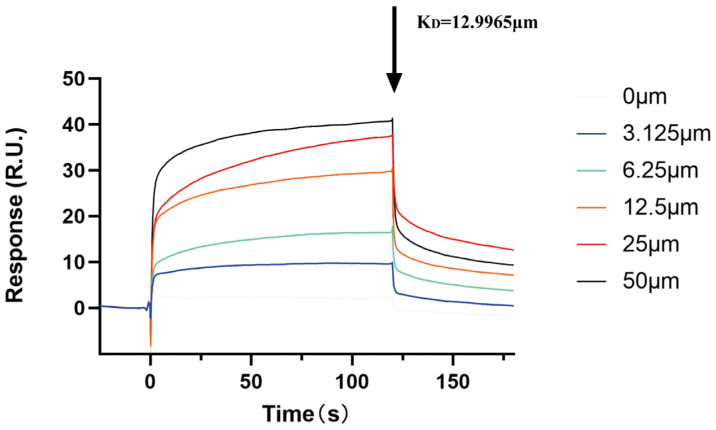
The binding of ACBA to HIF-1α is depicted through a surface plasmon resonance (SPR) sensorgram. The black arrow represents the beginning of ACBA dissociation from HIF-1α.

Finally, based on SPR technology, we used the Biacore T200 instrument (Cytiva, Uppsala, Sweden) to validate the specificity of the ACBA and HIF-1α interaction in vitro. The results demonstrated that the equilibrium dissociation constant between ACBA and HIF-1α was 12.9965 μM. Although ACBA solutions at various concentrations can bind to the HIF-1α protein, the intensity of the signal response increases with an increase in ACBA solution concentration. (Figure 10).

### 2.9. DMOG Antagonized the Inhibitory Effect of ACBA on M1 Polarization in LPS-Induced THP-1 Cells

**Figure 11:**
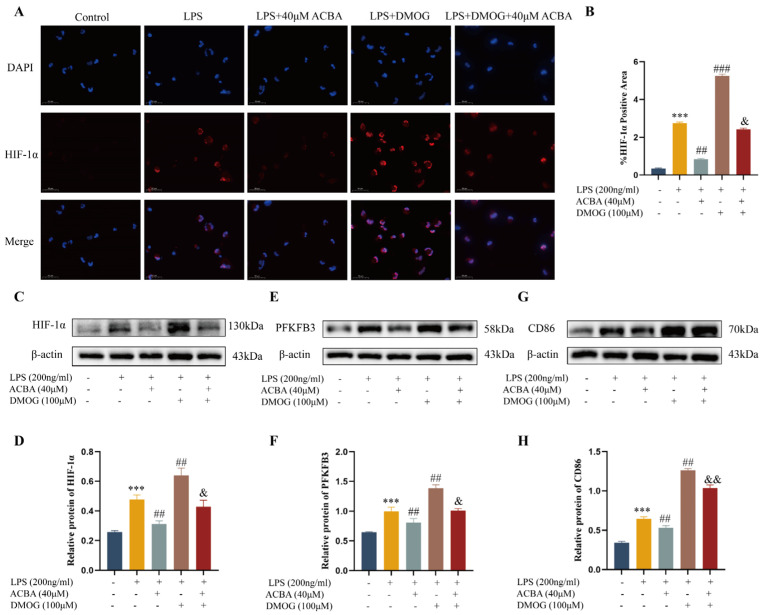
ACBA inhibits macrophage glycolysis and M1 polarization by targeting HIF-1α. (**A**) Immunofluorescence staining of HIF-1α in THP-1 cells. (**B**) Statistical analysis of HIF-1α-positive cells (*n* = 3). (**C**) Western blot analysis of HIF-1α. (**D**) Statistical analysis of HIF-1α protein expression levels (*n* = 3). (**E**) Western blot analysis of PFKFB3. (**F**) Statistical analysis of PFKFB3 protein expression levels (*n* = 3). (**G**) Western blot analysis of CD86. (**H**) Statistical analysis of CD86 protein expression levels (*n* = 3). Data are expressed as mean ± standard deviation. Compared to the control group, *** *p* < 0.001. Compared to the LPS group, ### *p* < 0.001 and ## *p* < 0.01. Compared to the LPS + ACBA group, && *p* < 0.01 and & *p* < 0.05.

We investigated whether DMOG, a HIF-specific agonist [31], could antagonize the effects of ACBA. Immunofluorescence analysis showed that DMOG significantly promoted the HIF-1α expression in LPS-induced THP-1 cells. Consistent with the above findings, 40 μM ACBA inhibited the HIF-1α expression. But compared to ACBA treatment alone, the combination of DMOG and ACBA treatment showed a significant upregulation of HIF-1α expression in LPS-induced THP-1 cells (Figure 11A,B). The Western blot results also revealed this specific expression pattern of HIF-1α (Figure 11C,D). Further, the expression of PFKFB3 and CD86 was analyzed by Western blot. Our results showed that ACBA significantly inhibited the PFKFB3 and CD86 expression in LPS-induced THP-1 cells, but DMOG eliminated the effect of ACBA (Figure 11E–H). These findings highlight that ACBA reduces macrophage glycolysis and M1 polarization by targeting HIF-1α.

### 2.10. ADMET Properties of ACBA

We predicted and analyzed the ADMET properties of ACBA by the Swiss ADME tool online. According to the standards, a chemical substance with drug-like properties must have a molecular weight (MW) below 500 Da, no more than five hydrogen bond donors (HBDs), fewer than ten hydrogen bond acceptors (HBAs), and a lipophilic log *p* value of less than 5 [32]. Table 2 presents the analysis of ACBA. The MW of ACBA is 444.47 Da, and the rotatable bonds (RBs) are 5, the HBA is 8, the HBD is 1, and the log *p* is 3.57. The estimated solubility (ESOL Log S) of ACBA is −5.23, and the estimated aqueous solubility (ESOL) is 5.93 × 10^−6^ mol/L, indicating that ACBA has good water solubility. The bioavailability score of ACBA is 0.55. The synthetic accessibility score (SAscore) of ACBA is 4.88. Additionally, ACBA exhibits high gastrointestinal absorption (GI) potential but weak blood–brain barrier (BBB) permeability.

## 3. Discussion

In recent years, medicinal plants and natural products have shown a variety of biological activities in anti-hepatic fibrosis, and their related drugs and active ingredients are considered a new research focus in the field of liver fibrosis prevention and treatment [33,34]. Heilaohu is a plant of the genus Schisandra in the family Schisandriaceae, distributed predominantly in Hunan, Jiangxi, and Guangxi provinces in China; known as “Dazuan” in Yao ethnomedicine, it was first officially documented in the Chinese pharmacopeia (1977 edition). In traditional Chinese medicine, apart from its fruit’s high nutritional value, Heilaohu is characterized by a warm, pungent, and slightly bitter taste, which has the effects of activating blood circulation, resolving stasis, and relieving flatulence. It is often used to treat rheumatoid arthritis, hepatitis, cirrhosis, and other diseases [17,35]. ACBA is one of the natural products extracted from the roots of Heilaohu and belongs to the biphenylcyclooctadiene lignan class of compounds [36]. In a previous study, we demonstrated firstly that ACBA alleviates CCl_4_-induced hepatic fibrosis in mice, but its specific targets and underlying mechanisms remain unclear [22]. In this study, the results showed that ACBA treatment markedly attenuated CCl_4_-induced inflammatory injury and fibrosis progression in mice hepatic tissues (Figure 1), which provided a basis for subsequent mechanistic research.

Liver macrophages are mainly classified as resident Kupffer cells (KCs) and monocyte-derived macrophages (MoMFs) according to their origin. As a liver’s innate immunological barrier, KCs preserve hepatic homeostasis and trigger the innate and adaptive immune responses. In contrast, MoMFs are recruited into the sites of inflammation during liver injury and differentiate into different phenotypic macrophages [37]. M1 macrophages represent high expression of CD86, CD68, and iNOS, and secrete the pro-inflammatory factors such as TNF-α, IL-6, and ROS, that exacerbate the hepatic inflammation and tissue injury and promote the development of hepatic fibrosis. Conversely, M2 macrophages highly express CD163 and CD206, and secrete anti-inflammatory factors such as IL-10 and TGF-β to participate in wound healing and tissue remodelling [38]. Thus, regulating the polarisation and dynamic balance of the M1/M2 phenotype is an effective treatment method for alleviating or even reversing hepatic fibrosis [39]. In this study, the ACBA treatment inhibited the expression of CD86 but promoted the expression of CD163 in CCl_4_-induced mouse liver tissues (Figure 2). Furthermore, the ACBA decreased the expression of CD86 and production of pro-inflammatory factors, as well as the proportion of CD86-positive macrophages, in LPS-induced THP-1 cells (Figure 3). The results suggest that ACBA inhibits M1 macrophage polarization and pro-inflammatory functions in mice with CCl_4_-induced hepatic fibrosis, which may be a key mechanism underlying ACBA’s therapeutic effects against hepatic fibrosis.

With the phenotype transition of macrophages, the cells’ metabolic patterns are also changed accordingly. Specifically, M1 macrophages primarily rely on glycolysis and the pentose phosphate pathway, whereas M2 macrophages depend on oxidative phosphorylation and enhanced fatty acid oxidation [40]. Thus, inhibition of glycolysis has been shown to suppress M1-polarized responses, including the production of ROS and secretion of pro-inflammatory cytokines [41,42]. For example, 6-phosphofructo-2-kinase/fructose-2,6-biphosphatase 3 (PFKFB3), as a key activator of phosphofructokinase 1 (PFK-1) in the process of glycolysis, promotes the phenotype of inflammatory macrophages, which is conducive to their uptake and elimination of virus-infected cells [43]. Conversely, the inhibitor of PFKFB3, such as 3PO, suppresses glycolytic activity, enhances oxidative phosphorylation, promotes M2 macrophage polarization, and downregulates the pro-inflammatory cytokines, thereby alleviating the tissue inflammation [44]. In the present study, ACBA treatment significantly reduced the expression of PFKFB3 in the CCl_4_-induced mice hepatic fibrosis tissues (Figure 4). Also, ACBA administration markedly downregulated the expression of PFKFB3 in LPS-induced THP-1 cells. Moreover, ACBA treatment can reduce the extracellular acidification rate (ECAR) and increase the oxygen consumption rate (OCR) of LPS-induced THP-1 cells (Figure 5). These findings suggest that ACBA inhibits the glycolytic metabolism of macrophages, thereby regulating the phenotype and function of hepatic macrophages.

Accumulating evidence has indicated that HIF-1α-drived enhancement of glycolytic fluxes promotes the M1 macrophage polarization and pro-inflammatory function [45,46,47]. Conversely, inhibiting HIF-1α-mediated glycolysis has been shown to decrease the M1 macrophage polarization and mitigate tissue inflammation [48,49]. Previous studies demonstrated that HIF-1α knockdown improved liver function and downregulated the expression of fibrosis-related genes in a bile duct ligation-induced hepatic fibrosis model [50]. Furthermore, HIF-1α-blocking reduced the pro-fibrotic factors secretion in hepatic Kupffer cells (KCs) under hypoxic conditions [51]. A recent report indicated there are increased levels of HIF-1α and TGF-β1 in peripheral blood mononuclear cells (PBMCs) and serum in HIV-infected individuals, and the upregulation of HIF-1α contributes to TGF-β-mediated HBV-induced liver fibrosis [52]. Additionally, studies report that HIF-1α mediates myeloid macrophage polarization via PFKFB3 and promotes renal fibrosis [53]. Thus, an immoderate HIF-1α regulates the M1 macrophage polarization through glycolytic pathways, which is one of the pivotal mechanisms for liver fibrosis. In the present study, we observed that ACBA treatment significantly attenuated CCl_4_-induced HIF-1α expression in hepatic tissues (Figure 6). Consistent with the in vivo findings, the ACBA treatment significantly downregulated the expression of HIF-1α levels in LPS-induced THP-1 cells (Figure 7). Based on these findings, we propose the potential mechanism for ACBA’s effects: it decreases the macrophages’ glycolysis through inhibiting HIF-1α, thereby regulating M1 macrophage polarization, contributing to alleviating liver inflammation and fibrosis.

In search of structural features of ACBA essential for HIF-1α inhibitory activity, we have successfully performed molecular docking and molecular dynamics simulations. Molecular docking results showed that ACBA interacts with three residues of the HIF-1α amino acid fragment 249–299 through hydrogen bonding and π-π stacking, which are precisely located within the PAS-B domain (Figure 8). Similarly, in a study of a potential HIF-1α inhibitor, the drug nilotinib—which binds to HIF-1α with high affinity—was found to have amino acid residues that form hydrogen bonds with HIF-1α also present in the PAS domain [54]. Further, molecular dynamics simulations demonstrated that the ACBA-HIF-1α complex maintains high structural stability. The Rg results indicate that the protein structure progressively attained a more compact state in the simulation process, also the SASA of the complex remained largely steady. In addition, the RMSD remained low throughout the simulation, implying minimal system deviation and the absence of major conformational changes in the molecules (Figure 9). Notably, our study revealed a peak with an RMSF value of 6Å near amino acid 350. We hypothesize that this occurs because the residue is located at the C-terminal of the PAS-B domain in HIF-1 protein, where the lack of a dense hydrogen bond network confers high flexibility. A similar phenomenon was also observed in Y. Singh’s research [55]. Then, using SPR technology, we confirmed the binding of ACBA to HIF-1α in vitro (Figure 10). The results suggest that the interaction between ACBA and HIF-1α involves multiple binding sites, and their interaction remains very stable within the appropriate range of ACBA concentrations. Finally, we discovered that DMOG treatment counteracted the inhibitory effect of ACBA on the expression of HIF-1α, PFKFB3, and CD86 in LPS-induced THP-1 cells (Figure 11). Moreover, the ADMET analysis results indicate that ACBA exhibits favorable drug-like properties (Table 2), positioning it as a promising candidate for further drug development. In summary, these findings indicate that ACBA binds to the PAS-B domain and destabilizes HIF-1α, thereby inhibiting its activation and transcriptional activity, which provides invaluable insights for developing natural drug molecules targeting macrophages for anti-fibrotic therapy.

However, this study still has several limitations that require consideration. Our results have revealed that ACBA alleviates CCl_4_-induced hepatic fibrosis in mice by inhibiting HIF-1α-mediated macrophage polarization, but the exact binding sites and patterns between ACBA and HIF-1α need to be validated in subsequent research. Additionally, comparative studies between HIF-1α natural antagonists and ACBA are still required. Finally, the long-term safety, effective dosage, and pharmacokinetic characteristics of ACBA in clinical applications need further evaluation in future studies.

## 4. Materials and Methods

### 4.1. Chemicals and Reagents

CCl_4_ (Macklin, Shanghai, China, #C805325), LPS (Solarbio, Beijing, China, #L8880), ACBA (Chengdu Pusi Biotechnology Co., Ltd., Chengdu, China, #PS3314-0005), α-SMA (AiFang Biological, Changsha, China, #AF10988), HRP-conjugated Goat Anti-Rabbit IgG secondary antibody (AiFang Biological, Changsha, China, #AFIHC003), HIF-1α (Uping Bio, Shenzhen, China, #YP-mAb-01776; Bioss, Beijing, China, #bs-0737R), PFKFB3 (Bioss, bsm-61693R; Proteintech, Wuhan, China, #13763-1-AP), CD86 (Santa Cruz, CA, USA, #sc-28347; Immunoway, San Jose, CA, USA, #YM8023), CD163 (Selleck, Houston, TX, USA, #F1548; Santa Cruz, #sc-58965; Abcam, Cambridge, UK, #ab182422), β-actin (Proteintech, Wuhan, China, #66009-1-IG), β-tubulin (Proteintech, Wuhan, China, #10094-1), HRP-conjugated secondary antibodies (Elabscience, Wuhan, China, #E-AB-1001, #E-AB-1003), APC anti-human CD86 Antibody (BioLegend, San Diego, CA, USA, #374207), Fluoroshield™ Mounting Medium with DAPI (Sigma, St. Louis, MO, USA, #F6057), HIF-1α protein (MCE, Junction, NJ, USA, #HY-P74888), CM5 chip (Cytiva, Uppsala, Sweden, #29104988), DMOG (MCE, Junction, NJ, USA, #HY-15893), ELISA kits (Elabscience, Wuhan, China, #E-EL-M3063, #E-EL-M0044c, #E-BC-235-M, #E-BC-K236-M), Seahorse XF Glycolysis Stress Test Kit (Agilent, Santa Clara, CA, USA, #103020-100), Amine Coupling Kit (Cytiva, Uppsala, Sweden, #BR100050).

### 4.2. Animal Experiments

Male C57BL/6 mice (aged 6–8 weeks, approximately 20 g) were procured from Hunan SJA Laboratory Animal Co., Ltd. in Changsha, Hunan, China, and raised at the Laboratory Animal Center of Hunan University of Traditional Chinese Medicine (license number: SYXK 2019-0009). The mice were divided into four groups: a control group, a model group, an ACBA high-dose treatment group (20 mg/kg) and a low-dose treatment group (5 mg/kg). Each group consisted of 10 mice. The control group was given daily intraperitoneal injections of an equivalent volume of normal saline. The model group and the ACBA treatment groups were administered intraperitoneal injections of 20% CCl_4_ in olive oil at 10 mL/kg twice weekly, and the ACBA treatment groups additionally received daily intraperitoneal injections of ACBA. All treatments were administered for four consecutive weeks.

### 4.3. Cell Culture

THP-1 cells were purchased from Procell (Wuhan, China) and cultured in RPMI-1640 medium supplemented with 10% fetal bovine serum and 1% penicillin/streptomycin at 37 °C in a 5% CO_2_ incubator. After differentiation with 100 ng/mL PMA for 24 h, the THP-1 cells were stimulated with 200 ng/mL LPS for 48 h. The cells were treated with high-dose ACBA (40 μmol/L), low-dose ACBA (10 μmol/L), DMOG (100 μmol/L), and a combination of DMOG and ACBA (DMOG 100 μmol/L, ACBA 40 μmol/L). An equal volume of basal medium was used for the control group.

### 4.4. H&E and Masson’s Trichrome Staining

Fresh hepatic tissue samples were fixed in 4% paraformaldehyde for 24 h. The samples were paraffin-embedded and sectioned at 5 μm. H&E staining of sections was performed using a fully automated H&E staining machine (Leica HistoCore SPECTRA ST, Wetzlar, Germany). Masson’s trichrome staining was performed according to the kit instructions (Servicebio, Wuhan, China, #cr2108024). All stained sections were imaged using the 3DHISTECH digital slide scanner system (Pannoramic MIDI, Budapest, Hungary) and analyzed with ImageJ 1.52a software.

### 4.5. Immunohistochemistry

The paraffin sections of hepatic tissue were dewaxed using xylene, then antigen repair via microwave treatment with citric acid buffer (PH 6.0). Endogenous peroxidase was blocked with 3% H_2_O_2_ oxidized at room temperature for 10 min. The sections were washed three times with PBST for 5 min each time, blocked with 5% goat serum for 1 h. The sections were incubated with α-SMA (1:200) and Collagen I (1:500) overnight at 4 °C, and incubated with HRP-conjugated Goat Anti-Rabbit IgG secondary antibody at room temperature for 1 h. All stained sections underwent image acquisition using the 3DHISTECH digital slide scanner system and analysis with ImageJ 1.52a software.

### 4.6. Western Blot

The fresh hepatic tissue or cultured cells were lysed using RIPA buffer supplemented with protease and phosphatase inhibitors. Protein quantification was performed using the BCA method. The extracted proteins were separated by SDS-PAGE and transferred to PVDF membranes. The membranes were blocked with 5% nonfat dry milk at room temperature for 2 h and incubated overnight at 4 °C with primary antibodies against Collagen I (1:500), α-SMA (1:5000), HIF-1α (1:1000), PFKFB3 (1:1000), CD86 (1:250), CD163 (1:1000), β-actin (1:40,000), and β-tubulin (1:5000). HRP-conjugated secondary antibodies (1:10,000) were incubated for 1 h at room temperature. Bands were visualized using the ChemiDOC XRS+ imaging system, and the gray values of target bands were analyzed with ImageLab 6.1 software.

### 4.7. ELISA

Mice serum or cell-culture supernatants were collected and analyzed by ELISA kits for the levels of TNF-α, IL-6, as well as the levels of ALT and AST. The manufacturer’s instructions were followed for the ELISA protocol.

### 4.8. Immunofluorescence

THP-1 cells cultured on coverslips were fixed with 95% ethanol for 30 min, blocked with 5% goat serum at room temperature for 30 min, and incubated with primary antibodies against CD86 (1:100), CD163 (1:100), HIF-1α (1:500), and PFKFB3 (1:500) overnight at 4 °C. The coverslips were subsequently incubated with Goat Anti-Rabbit IgG fluorescent secondary antibody (1:1000) at room temperature for 1 h. The slides were mounted using Fluoroshield™ Mounting Medium with DAPI. All coverslips were imaged using the 3DHISTECH digital slide scanner system and analyzed with ImageJ software.

The paraffin sections of hepatic tissue from each group were dewaxed using xylene, followed by antigen repair via microwave treatment with citric acid buffer (pH 6.0). The sections were blocked with 5% goat serum for 1 h, and incubated with primary antibodies CD86 (1:500), CD163 (1:500), HIF-1α (1:500), and PFKFB3 (1:500) overnight at 4 °C. The sections were incubated with HRP-conjugated Goat Anti-Rabbit IgG secondary antibody at room temperature for 1 h. All stained sections underwent image acquisition using the 3DHISTECH digital slide scanner system and analysis with ImageJ software.

### 4.9. RNA Extraction and RT-qPCR

The Total RNA Kit was used to extract total RNA from the fresh hepatic tissue according to the instructions. The RNA concentration was measured using a Nanodrop quantifier. Reverse transcription was performed with 1 μg total RNA using the reverse transcription system (Novoprotein, Suzhou, China, E047, E096). The Genesy 96T PCR system was used for real-time PCR for a 20 μL reaction system. The RT-PCR primer sequences are listed in Table 3.

### 4.10. Flow Cytometry

The differentiated THP-1 cells were collected and treated with FcR block. After incubation at 4 °C for 10 min, the cells were stained with APC anti-human CD86 Antibody at 4 °C for 30 min, then washed three times with PBS and fixed at room temperature for 30 min. Centrifuged at 150× *g* for 5 min and discarded the fixative. The cells were resuspended in 2 mL with 10× Intracellular Staining Permeabilization Wash Buffer, and centrifuged at 150× *g* for 5 min again. After resuspending in 500 μL cell staining buffer, the percentage of CD86-positive cells was analyzed using a flow cytometer.

### 4.11. Real-Time Cell Metabolism Assay

Treated cells were incubated in Seahorse XF Basal Medium containing 10 mmol/L glucose, 1 mmol/L pyruvate, and 2 mmol/L glutamine at room temperature for 1 h, and ECAR was measured with glycolytic and maximal glycolytic conditions by the Seahorse XF Energy Metabolism Analyzer. In OCR measurement, the cells were incubated at room temperature for 1 h in Seahorse XF Basal Medium containing 2 mmol/L glutamine, then the OCR was detected by the Seahorse XF Energy Metabolism Analyzer with basal respiration and maximal respiration conditions.

### 4.12. Molecular Docking

The 3D structures of HIF-1α (PDB ID: 4H6J) were obtained from the RCSB PDB database. The protein structure was processed using Pymol by removing water molecules and heteroatoms, and then hydrogenation was performed using the AutoDock Vina software. The 2D structure of ACBA was retrieved from the PubChem database, and energy minimization was performed using the Chem3D Ultra 14.0 software to obtain a stable 3D conformation. We set all rotatable bonds of ACBA to be flexible in AutoDock Tools. The search grid was centered on the HIF-1α native ligand pocket, with the following dimensions: center_x = 18.191 Å, center_y = −11.475 Å, center_z = −22.162 Å, size_x = 36 Å, size_y = 50 Å, size_z = 43 Å, and energy range = 5 kcal mol−1. For each system, twenty independent runs were performed; the pose with the lowest affinity score was analyzed in Pymol. Binding modes were visualized and analyzed for hydrogen bonds and π-π stacking using Pymol and Discovery Studio Visualizer.

### 4.13. Molecular Dynamics Simulations

GROMACS 2023 software was used for molecular dynamics simulation to further analyze the stability of complexes formed between ACBA and HIF-1α. The force field parameters are generated using GROMACS’s pdb2gmx tool. The GAFF2 force field topology file was created using sobtop_1.0 (dev3.1) software. RESP was applied to distribute the charge of the ligand, and the AMBER14SB force field parameters were utilized for the receptor. During simulations, the TIP3P water model was employed for system solvation, with a 1 nm cubic water box. A solution with 0.15 M concentration of NaCl was added by GROMACS’s gmx genion tool to maintain electrical neutrality. Long-range electrostatic interactions are treated using the Particle Mesh Ewald (PME) method, with a 1 nm cut-off radius. Energy minimization was performed using the steepest descent method and conjugate gradient optimization. The LINCS algorithm was employed to bond constraints. The temperature was maintained at 310 K using the Nose–Hoover thermostat, while pressure was controlled at 1 bar via the Parrinello–Rahman barostat. Subsequently, equilibration was performed under NPT ensembles. The simulation duration was 100 ns with an integration time step of 2 fs. The gyration radius (Rg) and solvent-accessible surface area (SASA), root mean square deviations (RMSDs) and root mean square fluctuations (RMSFs) were calculated using the built-in “gmx_Rg”, “gmx_sasa”, “gmx_rmsd” and “gmx_rmsf” functions in the GROMACS 2023 software.

### 4.14. SPR Assay

SPR was performed using the Biacore T200 instrument. The Amine Coupling Kit was utilized to activate the CM5 chip, followed by the immobilization of recombinant protein HIF-1α to the chip surface. Five concentration gradients of ACBA (3.125, 6.25, 12.5, 25, and 50 μmol/L) were injected into the Biacore system at an injection time of 60 s and a flow rate of 30 μL/min. Response values were observed from the output sensor plot results.

### 4.15. ADMET Properties Prediction

The Swiss ADME tool was applied to determine the pharmacokinetic characteristics [56]. We predicted the pharmacokinetic characteristics of ACBA, including molecular weight (MW), rotatable bonds (RBs), hydrogen bond acceptors (HBAs), hydrogen bond donors (HBDs), log P, ESOL Log S, ESOL (mol/L), bioavailability score, synthetic accessibility score (SAscore), gastrointestinal (GI) absorption, blood–brain barrier (BBB) permeability, and P-glycoprotein substrate, with the tool online. ACBA was obtained by using the PubChem database.

### 4.16. Statistical Analysis

All data are expressed as mean ± standard deviation. The statistical differences between the two groups were analyzed using a two-tailed, unpaired Student’s *t*-test. Differences between data from multiple groups were analyzed using one-way analysis of variance (ANOVA) with Bonferroni correction (GraphPad Prism 9.0, San Diego, CA, USA). Statistical significance was defined as *p* < 0.05.

## 5. Conclusions

In summary, this study elucidated the mechanism by which ACBA alleviates CCl_4_-induced liver fibrosis in mice. ACBA significantly reduces the M1 macrophage phenotype polarization in fibrotic liver tissue. Both in vivo and in vitro experiments confirmed that ACBA inhibits HIF-1α expression and glycolysis in macrophages. Molecular docking revealed three potential binding sites for ACBA within the PAS-B domain of HIF-1α: the amino acid residues SER-274, Tyr-276, and HIS-291. Molecular dynamics simulations demonstrated stable binding between ACBA and HIF-1α. Further in vitro experiments showed that ACBA, by targeting HIF-1α, suppresses macrophage glycolysis and M1 macrophage polarization. Our findings provided critical evidence for the clinical application of ACBA in anti-fibrotic therapy and highlight its potential as a novel therapeutic agent.

## Figures and Tables

**Table 1:** Binding affinity rank of ACBA and HIF-1α in twenty independent runs.

Mode	Affinity (kcal/mol)	Rmsd l.b.	Rmsd u.b.
1	−6.0	0.000	0.000
2	−5.874	1.745	6.466
3	−5.867	25.61	27.99
4	−5.797	15.84	18.73
5	−5.538	6.878	10.39
6	−5.508	24.58	26.76
7	−5.425	5.503	9.721
8	−5.42	20.29	23.51
9	−5.389	25.48	27.47
10	−5.277	16.24	19.54
11	−5.27	19.63	22.73
12	−5.242	9.833	13.6
13	−5.139	19.31	22.29
14	−5.127	25.21	27.62
15	−5.104	4.821	8.209
16	−5.092	25.64	27.09
17	−5.06	21.89	23.67
18	−4.997	19.92	22.06
19	−4.997	19.52	22.93
20	−4.988	11.52	15.57

**Table 2:** The analysis of the pharmacokinetics and pharmacodynamics properties of ACBA.

Molecules	MW	RB	HBA	HBD	Log *p*	ESOL Log S	ESOL (mol/L)	Bioavailability Score	P-gp Substrate	SA Score	GI	BBB
ACBA	444.47	5	8	1	3.57	−5.23	5.93 × 10^−6^	0.55	No	4.88	High	No

**Table 3:** Primer sequences for real-time RT-PCR.

Gene	Forward Primer (5′–3′)	Reverse Primer (5′–3′)
CD86	TGGGCTTGGCAATCCTTATCTT	CCAGCTCACTCAGGCTTATGTTT
CD163	AGGAAACCAATCCCAGACACTA	CGACCACCTCCACCTACCAA
HIF-1α	AACCCATTCCTCATCCGTCAA	TTCAACCCAGACATATCCACCTC
β-actin	GTGACGTTGACATCCGTAAAGA	GTAACAGTCCGCCTAGAAGCAC

## Data Availability

Data are contained within the article.

## Abbreviations

The following abbreviations are used in this manuscript:

HF	Hepatic fibrosis
HIF-1α	Hypoxia inducible factor-1α
ACBA	Acetylbinankadsurin A
CCl_4_	Carbon tetrachloride
SPR	Surface plasmon resonance
ALT	Alanine aminotransferase
AST	Aspartate aminotransferase
TNF-α	Tumor necrosis factor-alpha
PFKFB3	6-phosphofructo-2-kinase/fructose-2,6-biphosphatase 3
ECARs	Extracellular acidification rates
OCRs	Oxygen consumption rates
DMOG	Dimethyloxallyl glycine
HSCs	Hepatic stellate cells
ECM	Extracellular matrix
KCs	Kuffer cells
Rg	Radius of gyration
SASA	Solvent-accessible surface area
RMSD	Root mean square deviation
RMSF	Root mean square fluctuation
KD	Equilibrium dissociation constant
MoMFs	Monocyte-derived macrophages
PAS	Periodicity-ARNT-Single-minded
MW	Molecular weight
RBs	Rotatable bonds
HBAs	Hydrogen bond acceptors
HBDs	Hydrogen bond donors
ESOL Log S	Estimated solubility
ESOL	Estimated aqueous solubility
SAscore	Synthetic accessibility score
GI	Gastrointestinal absorption
BBB	Blood–brain barrier

## References

[1] Asrani S.K., Devarbhavi H., Eaton J., Kamath P.S. (2019). Burden of Liver Diseases in the World. J. Hepatol..

[2] Taru V., Szabo G., Mehal W., Reiberger T. (2024). Inflammasomes in Chronic Liver Disease: Hepatic Injury, Fibrosis Progression and Systemic Inflammation. J. Hepatol..

[3] Chen L., Guo W., Mao C., Shen J., Wan M. (2024). Liver Fibrosis: Pathological Features, Clinical Treatment and Application of Therapeutic Nanoagents. J. Mater. Chem. B.

[4] Roehlen N., Crouchet E., Baumert T.F. (2020). Liver Fibrosis: Mechanistic Concepts and Therapeutic Perspectives. Cells.

[5] Cheng D., Chai J., Wang H., Fu L., Peng S., Ni X. (2021). Hepatic Macrophages: Key Players in the Development and Progression of Liver Fibrosis. Liver Int..

[6] Wang Z., Du K., Jin N., Tang B., Zhang W. (2023). Macrophage in Liver Fibrosis: Identities and Mechanisms. Int. Immunopharmacol..

[7] van der Heide D., Weiskirchen R., Bansal R. (2019). Therapeutic Targeting of Hepatic Macrophages for the Treatment of Liver Diseases. Front. Immunol..

[8] Wang W., Li S., Liu Y., Ding X., Yang Y., Chen S., Cao J., Tacke F., Dong W., Lan T. (2025). Macrophage Heterogeneity in Liver Fibrosis. Front. Immunol..

[9] Kierans S.J., Taylor C.T. (2021). Regulation of Glycolysis by the Hypoxia-Inducible Factor (HIF): Implications for Cellular Physiology. J. Physiol..

[10] Tawakol A., Singh P., Mojena M., Pimentel-Santillana M., Emami H., MacNabb M., Rudd J.H.F., Narula J., Enriquez J.A., Través P.G. (2015). HIF-1α and PFKFB3 Mediate a Tight Relationship Between Proinflammatory Activation and Anerobic Metabolism in Atherosclerotic Macrophages. Arterioscler. Thromb. Vasc. Biol..

[11] Koyasu S., Kobayashi M., Goto Y., Hiraoka M., Harada H. (2018). Regulatory Mechanisms of Hypoxia-Inducible Factor 1 Activity: Two Decades of Knowledge. Cancer Sci..

[12] Taylor C.T., Scholz C.C. (2022). The Effect of HIF on Metabolism and Immunity. Nat. Rev. Nephrol..

[13] Wu D., Potluri N., Lu J., Kim Y., Rastinejad F. (2015). Structural Integration in Hypoxia-Inducible Factors. Nature.

[14] Zhang J., Yao M., Xia S., Zeng F., Liu Q. (2025). Systematic and Comprehensive Insights into HIF-1 Stabilization under Normoxic Conditions: Implications for Cellular Adaptation and Therapeutic Strategies in Cancer. Cell. Mol. Biol. Lett..

[15] Chen S., Wu Z., Zhang J., Lin Y., Xie J., Yin D., Zhu Y. (2025). Research and Application of Medicines for Treating Liver Fibrosis: Current Status and Prospects. Front. Pharmacol..

[16] Kuete J.B.T., Kuete J.R.N., Mbaveng A.T., Kuete V., Efferth T., Weiskirchen R. (2025). A Three-Year Review (2023–2025) on the Effectiveness of Natural Products from Plants in Treating Major Types of Fibrosis. Phytomedicine.

[17] Long H., Xia X., Liao S., Wu T., Wang L., Chen Q., Wei S., Gu X., Zhu Z. (2022). Physicochemical Characterization and Antioxidant and Hypolipidaemic Activities of a Polysaccharide from the Fruit of *Kadsura coccinea* (Lem.) A. C. Smith. Front. Nutr..

[18] Jia Y.-Z., Yang Y.-P., Cheng S.-W., Cao L., Xie Q.-L., Wang M.-Y., Li B., Jian Y.-Q., Liu B., Peng C.-Y. (2021). Heilaohuguosus A-S from the Fruits of *Kadsura coccinea* and Their Hepatoprotective Activity. Phytochemistry.

[19] Yang Y., Jian Y., Cheng S., Jia Y., Liu Y., Yu H., Cao L., Li B., Peng C., Iqbal Choudhary M. (2021). Dibenzocyclooctadiene Lignans from *Kadsura coccinea* Alleviate APAP-Induced Hepatotoxicity via Oxidative Stress Inhibition and Activating the Nrf2 Pathway in Vitro. Bioorg. Chem..

[20] Wang Y.-S., Liu S.-Q., Yao Y.-X., Zhang S., Li D.-H., Li H.-Y., Yu H.-H., Yuan H.-W., Wang W., Li B. (2025). Integrated Network Pharmacology and Metabolomics to Explore the Mechanism of *Kadsura coccinea* Root in Alleviating Acetaminophen-Induced Liver Injury. J. Ethnopharmacol..

[21] Liu Y., Yang Y., Tasneem S., Hussain N., Daniyal M., Yuan H., Xie Q., Liu B., Sun J., Jian Y. (2018). Lignans from Tujia Ethnomedicine Heilaohu: Chemical Characterization and Evaluation of Their Cytotoxicity and Antioxidant Activities. Molecules.

[22] Peng W., Yang Y., Lu H., Shi H., Jiang L., Liao X., Zhao H., Wang W., Liu J. (2024). Network Pharmacology Combines Machine Learning, Molecular Simulation Dynamics and Experimental Validation to Explore the Mechanism of Acetylbinankadsurin A in the Treatment of Liver Fibrosis. J. Ethnopharmacol..

[23] Sakai M., Troutman T.D., Seidman J.S., Ouyang Z., Spann N.J., Abe Y., Ego K.M., Bruni C.M., Deng Z., Schlachetzki J.C.M. (2019). Liver-Derived Signals Sequentially Reprogram Myeloid Enhancers to Initiate and Maintain Kupffer Cell Identity. Immunity.

[24] Chi G., Pei J., Li X. (2023). The Imbalance of Liver Resident Macrophages Polarization Promotes Chronic Autoimmune Hepatitis Development in Mice. PeerJ.

[25] Ma P.-F., Gao C.-C., Yi J., Zhao J.-L., Liang S.-Q., Zhao Y., Ye Y.-C., Bai J., Zheng Q.-J., Dou K.-F. (2017). Cytotherapy with M1-Polarized Macrophages Ameliorates Liver Fibrosis by Modulating Immune Microenvironment in Mice. J. Hepatol..

[26] Liu Q., Li J., Li X., Zhang L., Yao S., Wang Y., Tuo B., Jin H. (2024). Advances in the Understanding of the Role and Mechanism of Action of PFKFB3-mediated Glycolysis in Liver Fibrosis (Review). Int. J. Mol. Med..

[27] Schmidt C.A., Fisher-Wellman K.H., Neufer P.D. (2021). From OCR and ECAR to Energy: Perspectives on the Design and Interpretation of Bioenergetics Studies. J. Biol. Chem..

[28] Minchenko A., Leshchinsky I., Opentanova I., Sang N., Srinivas V., Armstead V., Caro J. (2002). Hypoxia-Inducible Factor-1-Mediated Expression of the 6-Phosphofructo-2-Kinase/Fructose-2,6-Bisphosphatase-3 (PFKFB3) Gene. Its Possible Role in the Warburg Effect. J. Biol. Chem..

[29] Wang X., Liu X., Wu W., Liao L., Zhou M., Wang X., Tan Z., Zhang G., Bai Y., Li X. (2024). Hypoxia Activates Macrophage-NLRP3 Inflammasome Promoting Atherosclerosis via PFKFB3-Driven Glycolysis. FASEB J..

[30] Liu Y., He C.-Y., Yang X.-M., Chen W.-C., Zhang M.-J., Zhong X.-D., Chen W.-G., Zhong B.-L., He S.-Q., Sun H.-T. (2023). Paeoniflorin Coordinates Macrophage Polarization and Mitigates Liver Inflammation and Fibrogenesis by Targeting the NF-[Formula: See Text]B/HIF-1α Pathway in CCl4-Induced Liver Fibrosis. Am. J. Chin. Med..

[31] Nowak-Stępniowska A., Osuchowska P.N., Fiedorowicz H., Trafny E.A. (2022). Insight in Hypoxia-Mimetic Agents as Potential Tools for Mesenchymal Stem Cell Priming in Regenerative Medicine. Stem Cells Int..

[32] Noureddine O., Issaoui N., Al-Dossary O. (2021). DFT and Molecular Docking Study of Chloroquine Derivatives as Antiviral to Coronavirus COVID-19. J. King Saud. Univ. Sci..

[33] El-Tantawy W.H., Temraz A. (2022). Anti-Fibrotic Activity of Natural Products, Herbal Extracts and Nutritional Components for Prevention of Liver Fibrosis: Review. Arch. Physiol. Biochem..

[34] Li J.-Z., Chen N., Ma N., Li M.-R. (2023). Mechanism and Progress of Natural Products in the Treatment of NAFLD-Related Fibrosis. Molecules.

[35] Yang Y.-P., Hussain N., Zhang L., Jia Y.-Z., Jian Y.-Q., Li B., Iqbal Choudhary M., Rahman A.-U., Wang W. (2020). *Kadsura coccinea*: A Rich Source of Structurally Diverse and Biologically Important Compounds. Chin. Herb. Med..

[36] Sun Q.-Z., Chen D.-F., Ding P.-L., Ma C.-M., Kakuda H., Nakamura N., Hattori M. (2006). Three New Lignans, Longipedunins A-C, from *Kadsura longipedunculata* and Their Inhibitory Activity against HIV-1 Protease. Chem. Pharm. Bull..

[37] Conte E. (2022). Targeting Monocytes/Macrophages in Fibrosis and Cancer Diseases: Therapeutic Approaches. Pharmacol. Ther..

[38] Ramachandran P., Iredale J.P., Fallowfield J.A. (2015). Resolution of Liver Fibrosis: Basic Mechanisms and Clinical Relevance. Semin. Liver Dis..

[39] Zhang J., Xie Z., Zhu X., Xu C., Lin J., Zhao M., Cheng Y. (2025). New Insights into Therapeutic Strategies for Targeting Hepatic Macrophages to Alleviate Liver Fibrosis. Int. Immunopharmacol..

[40] Viola A., Munari F., Sánchez-Rodríguez R., Scolaro T., Castegna A. (2019). The Metabolic Signature of Macrophage Responses. Front. Immunol..

[41] Freemerman A.J., Johnson A.R., Sacks G.N., Milner J.J., Kirk E.L., Troester M.A., Macintyre A.N., Goraksha-Hicks P., Rathmell J.C., Makowski L. (2014). Metabolic Reprogramming of Macrophages: Glucose Transporter 1 (GLUT1)-Mediated Glucose Metabolism Drives a Proinflammatory Phenotype. J. Biol. Chem..

[42] Pavlou S., Wang L., Xu H., Chen M. (2017). Higher Phagocytic Activity of Thioglycollate-Elicited Peritoneal Macrophages Is Related to Metabolic Status of the Cells. J. Inflamm..

[43] Jiang H., Shi H., Sun M., Wang Y., Meng Q., Guo P., Cao Y., Chen J., Gao X., Li E. (2016). PFKFB3-Driven Macrophage Glycolytic Metabolism Is a Crucial Component of Innate Antiviral Defense. J. Immunol..

[44] Zhao Y., Peng Y., Wang D., Zhang L., Qiu Y., Cui J., Xie F., Zhu N., Qin M., Wang Y. (2025). PFKFB3-Inhibitor 3PO-Mediated Glycolytic Reprogramming Promotes Inflammatory Dental Pulp Repair: An In Vitro and In Vivo Study. Int. Endod. J..

[45] Wu D., Xu J., Jiao W., Liu L., Yu J., Zhang M., Chen G. (2023). Suppression of Macrophage Activation by Sodium Danshensu via HIF-1α/STAT3/NLRP3 Pathway Ameliorated Collagen-Induced Arthritis in Mice. Molecules.

[46] Wu K.K.-L., Xu X., Wu M., Li X., Hoque M., Li G.H.Y., Lian Q., Long K., Zhou T., Piao H. (2024). MDM2 Induces Pro-Inflammatory and Glycolytic Responses in M1 Macrophages by Integrating iNOS-Nitric Oxide and HIF-1α Pathways in Mice. Nat. Commun..

[47] Zhang J., Yuan Z., Li X., Wang F., Wei X., Kang Y., Mo C., Jiang J., Liang H., Ye L. (2023). Activation of the JNK/COX-2/HIF-1α Axis Promotes M1 Macrophage via Glycolytic Shift in HIV-1 Infection. Life Sci. Alliance.

[48] Zeng X., Li T., Yang K., Jiang Y., Chen S., Yang S., Zou S., Liu J., Duan P. (2024). Natural Compound Phloretin Restores Periodontal Immune Homeostasis via HIF-1α-Regulated PI3K/Akt and Glycolysis in Macrophages. Int. Immunopharmacol..

[49] Lin Z.-J., Dong X., He H., Jiang J.-L., Guan Z.-J., Li X., Lu L., Li H., Huang Y.-S., Xian S.-X. (2024). A Simplified Herbal Decoction Attenuates Myocardial Infarction by Regulating Macrophage Metabolic Reprogramming and Phenotypic Differentiation via Modulation of the HIF-1α/PDK1 Axis. Chin. Med..

[50] Moon J.-O., Welch T.P., Gonzalez F.J., Copple B.L. (2009). Reduced Liver Fibrosis in Hypoxia-Inducible Factor-1alpha-Deficient Mice. Am. J. Physiol. Gastrointest. Liver Physiol..

[51] Copple B.L., Bai S., Moon J.-O. (2010). Hypoxia-Inducible Factor-Dependent Production of Profibrotic Mediators by Hypoxic Kupffer Cells. Hepatol. Res..

[52] Xu M., Warner C., Duan X., Cheng Z., Jeyarajan A.J., Li W., Wang Y., Shao T., Salloum S., Chen P.-J. (2024). HIV Coinfection Exacerbates HBV-Induced Liver Fibrogenesis through a HIF-1α- and TGF-Β1-Dependent Pathway. J. Hepatol..

[53] Yang Q., Huo E., Cai Y., Zhang Z., Dong C., Asara J.M., Shi H., Wei Q. (2023). Myeloid PFKFB3-Mediated Glycolysis Promotes Kidney Fibrosis. Front. Immunol..

[54] Reda D., Elfiky A.A., Elnagdy M., Khalil M.M. (2024). Molecular Docking and Molecular Dynamics of Hypoxia-Inducible Factor (HIF-1alpha): Towards Potential Inhibitors. J. Biomol. Struct. Dyn..

[55] Singh Y., Sanjay K.S., Kumar P., Singh S., Thareja S. (2023). Molecular Dynamics and 3D-QSAR Studies on Indazole Derivatives as HIF-1α Inhibitors. J. Biomol. Struct. Dyn..

[56] Salamat A., Kosar N., Mohyuddin A., Imran M., Zahid M.N., Mahmood T. (2024). SAR, Molecular Docking and Molecular Dynamic Simulation of Natural Inhibitors against SARS-CoV-2 Mpro Spike Protein. Molecules.

